# Exploring Breastfeeding Mothers’ and Lactation Consultants’ Experiences of Lactation Consultancy Throughout the Restrictions Put in Place Due to the COVID-19 Pandemic

**DOI:** 10.12688/hrbopenres.13856.2

**Published:** 2024-12-16

**Authors:** Anna Connolly, Anne Matthews

**Affiliations:** 1 Dublin City University, Dublin, Leinster, Ireland

**Keywords:** Breastfeeding, COVID-19, Breastfeeding Support, Lactation

## Abstract

**Background:**

Breastfeeding rates in Ireland are among the lowest in the world. Lactation consultancy provides mothers with support and information on how to cope with any challenges they encounter. There is emerging evidence that COVID-19 restrictions impacted access to and the quality of breastfeeding support.

The aim of this study was to explore breastfeeding mothers’ and lactation consultants’ experiences of breastfeeding support throughout the COVID-19 restrictions in Ireland. It also aimed to explore what adaptations had to be made to the delivery of lactation consultancy and how these changes impacted mothers’ experiences of breastfeeding support.

**Methods:**

A qualitative research design was chosen. Semi-structured interviews were conducted with eight participants, three breastfeeding mothers and five lactation consultants. Interviews were conducted online via Zoom, audio-recorded and transcribed. The data were analysed using thematic analysis.

**Results:**

Five key themes were identified: ‘Lack of Support’, ‘Adapting to COVID-19 Restrictions’, Emotional Response to COVID-19 and Restrictions’, Vaccination against SARS-CoV-2 as a Barrier to and Facilitator of Support’ and Inconsistency of Lactation Support Across Ireland’. Both lactation consultants and mothers identified similar issues however, slight variations within lactation consultants' perceptions of mothers’ and mothers’ attitudes towards online services were seen. An notable finding was the lack of support in hospitals pre-COVID-19.

**Conclusions:**

Both mothers’ and lactation consultants’ experiences of lactation consultancy were impacted by the COVID-19 restrictions. Although exacerbated by COVID-19, the lack of support in hospitals existed before COVID-19. Provision of better breastfeeding support is required.

Increased availability of lactation consultants and the implementation of breastfeeding rooms within hospitals is required in addition to debrief counselling sessions for lactation consultants. Further research is required to understand the unavailability of lactation consultants in hospital settings and to identify how to manage breastfeeding support in future emergency situations.

## List of abbreviations

WHO      World Health Organisation

NWIHP   National Women and Infants Health Programme

IBCLC    International Board Certified Lactation Consultant

PHN       Public Health Nurse

DoH       Department of Health

ALCI     Association of Lactation Consultants in Ireland

IT          Information Technology

NICU    Neonatal Intensive Care Unit

HSE      Health Service Executive

PPE       Personal Protective Equipment

## Introduction

Breastfeeding rates globally do not meet the WHO targets ( Global Breastfeeding Collective, 2021) and in Ireland they are among the lowest in Europe and the world ( Leahy-Warren *et al.*, 2017; NWIHP, 2021). According to most recent data available, only 15% of babies in Ireland are exclusively breastfed at six months old ( WHO, 2014). This rate is extremely low in comparison to the global rate of 44% ( Global Breastfeeding Collective, 2021). Breastfeeding is one of the most successful ways of ensuring child health and survival ( WHO, 2022) with benefits such as protection against infections, diarrhoea and diabetes. Breastfeeding may protect mothers against breast cancer, ovarian cancer and type 2 diabetes and improve birth spacing ( Victora *et al.*, 2016; WHO, 2022). Breastfeeding’s immunological benefits are being recognised in the context of COVID-19 ( Walker *et al.*, 2022). Breastmilk of women who received two doses of SARS-CoV-2 vaccine was found to contain antibodies which may offer protection against COVID-19 for infants ( Baird *et al.*, 2021). One-on-one time with lactation consultants and other healthcare professionals who provide breastfeeding support is significantly beneficial to breastfeeding mothers by providing them with useful information on how to cope with breastfeeding challenges they face, and therefore prolonging breastfeeding duration ( Alberdi *et al.*, 2018; Gavine *et al.*, 2022; Giltenane *et al.*, 2021; McGuinness *et al.*, 2020; Theurich *et al.*, 2019; Whelan & Kearney, 2015). The COVID-19 restrictions hugely impacted access to breastfeeding support with many women feeling frustrated, stressed and dissatisfied with insufficient video consultations with lactation consultants ( Panda *et al.*, 2021). The rate of babies that had received any breastfeeding on discharge from hospital dropped 4.9 percentage points between 2019 and 2020 in Ireland. Only 58.5% of babies had received any type of breastfeeding on discharge from hospital in 2020 in comparison to 63.4% in 2019. It was suggested that this decline could be attributable to COVID-19 and the changes it caused in behaviour on post-natal hospital wards ( NWIHP, 2021). Further research was warranted to explore both mothers’ and lactation consultants’ experiences of lactation consultancy throughout the COVID-19 restrictions in Ireland.

The term lactation consultant will be frequently referred to. A lactation consultant can be defined as a healthcare professional that has specific training to deliver breastfeeding support and assist with the prevention, recognition and provision of solutions to breastfeeding difficulties ( Brooks *et al.*, 2016)

Given the benefits of breastfeeding, facilitating and supporting women to breastfeed is vital. The importance of support from healthcare professionals such as International Board Certified Lactation Consultants (IBCLC) and public health nurses (PHN) (in the Irish context), health visitors, community health nurses/midwives has been widely recognised given its association with positive breastfeeding outcomes such as increased initiation, duration and exclusivity rates ( Alberdi *et al.*, 2018; Gavine *et al.*, 2022; Leahy-Warren *et al.*, 2017; McGuinness *et al.*, 2020; North *et al.*, 2022; Whelan & Kearney, 2015). Women who attended consultations with IBCLCs and PHN led support groups felt that the support boosted their confidence and encouraged them to breastfeed ( Alberdi *et al.*, 2018; Giltenane *et al.*, 2021; Leahy-Warren *et al.*, 2017; McCarthy Quinn *et al.*, 2019; McGuinness *et al.*, 2020). Unfortunately, many women cease breastfeeding early due to the challenges they face however, support may help women overcome these issues and allow them to continue breastfeeding for longer ( Gavine *et al.*, 2022; McGuinness *et al.*, 2020). Previous studies have shown how beneficial support from healthcare professionals and breastfeeding support groups can be in providing women with the right tools and techniques to overcome their challenges ( Alberdi *et al.*, 2018; McCarthy Quinn *et al.*, 2019). Face-to-face support has been identified by mothers as important ( Ingram *et al.*, 2020), as with phone, digital or combined support methods, face-to-face support has been and shown to be more successful in encouraging women to continue and exclusively breastfeed ( Gavine *et al.*, 2022; World Health Organization, 2018). Although the importance of breastfeeding support is well documented, women in Ireland have reported receiving inconsistent, contradictory and poor quality information on breastfeeding in addition to limited availability of support on post-natal wards with little or no access to lactation consultants ( DOH, 2016).

Support is essential in facilitating breastfeeding and allowing its benefits to be experienced by both mothers and infants. However, the COVID-19 pandemic has presented challenges to breastfeeding and support services ( Brown & Shenker, 2021; Panda *et al.*, 2021). In December 2019, COVID-19, an infectious respiratory disease, emerged and resulted in significant restrictions on society. A new strain of coronavirus called SARS-CoV-2 is responsible for this disease which is transmitted through respiratory droplets ( Chakraborty & Maity, 2020). In March 2020, the Irish government imposed a lockdown where people were asked to remain at home. Since then, Ireland experienced a continuous tightening and easing of restrictions that saw general practitioners move their services online and pre and post-natal appointments be postponed ( Kennelly *et al.*, 2020). During the pandemic, face-to-face contact with PHNs and IBCLCs was curtailed, support groups were suspended and family support was restricted ( Kinoshita & Doolan, 2021; Lalor *et al.*, 2021; Panda *et al.*, 2021). Many mothers had difficulty accessing support and expressed their frustration over receiving ‘useless’ breastfeeding booklets and insufficient online consultations ( Panda *et al.*, 2021). A study conducted by Brown and Shenker (2021) in the UK found that 27% of mothers struggled to access support and ceased breastfeeding before they wanted to. Other challenges to breastfeeding and breastfeeding support included the separation of mothers from infants in hospitals due to fear of infection ( Gribble *et al.*, 2020) and personal protective equipment (PPE) such as masks that compromised communication between healthcare professionals and parents and parents and infants ( Green *et al.*, 2021).

In conclusion, breastfeeding rates in Ireland are low despite the clear benefits to both mothers and infants. Breastfeeding and breastfeeding supports have been challenged by COVID-19 and the restrictions. However, lactation support throughout the pandemic in an Irish context has not been previously explored from the perspective of both mothers and healthcare professionals. This provides clear rationale for research to be carried out combining both lactation consultants’ and breastfeeding mothers’ experiences of lactation consultancy in Ireland in the context of COVID-19.

## Methods

This research was conducted as part of an undergraduate degree programme. The research including the interviews and data analysis were carried out by the final year undergraduate student whose research project was informed by this study.

### Aim

The aim of this study was to explore breastfeeding mothers’ and lactation consultants’ experiences of breastfeeding support throughout the COVID-19 restrictions in Ireland. It also aimed to explore what adaptations had to be made to the delivery of lactation consultancy and how these changes impacted mothers’ experiences of breastfeeding support.

### Objectives

1. To explore the impact of COVID-19 restrictions on mothers’ access to lactation consultancy and breastfeeding support
2. To explore how the COVID-19 restrictions impacted the delivery of lactation consultancy
3. To investigate what adaptations have had to be made to the delivery of lactation consultancy due to COVID-19 restrictions
4. To explore how these changes have impacted mothers’ experiences of breastfeeding in Ireland during the COVID-19 pandemic.

### Research design

A qualitative research design was used as it provided insight and understanding into individuals’ experiences and allowed the voices of participants to be included in the research ( Denny & Weckesser, 2019). A quantitative research design places emphasis on numerical and statistical data ( Liamputtong & Rice, 2021) and therefore, was deemed inappropriate for this study. The qualitative data collection method was semi-structured interviews as they are valuable for collecting rich data based on experiences and perceptions and because verbal communication is a valuable way of conveying emotions, feelings, experiences and views ( Parahoo, 2014), which fits with the aim of this research study. Interviews were conducted with both lactation consultants and mothers to gain insight into the different perspectives that these groups have of lactation consultancy in Ireland throughout the COVID-19 restrictions.

### Sample

Participants were selected from target populations of breastfeeding mothers and lactation consultants. Purposively selecting a sample from a target population is appropriate as the data collection, processing and analysis are cost and time efficient ( Daniel, 2015). Sampling is a low-cost method that allows the researcher to collect the necessary data within the time constraints of the project. Purposive sampling was used to select participants who had first-hand experience of breastfeeding support who were able to provide rich and valuable data relevant to the topic being explored. A small sample size of eight participants ( Figure 1A) was used as this was large enough to generate original and high quality, rich data but also small enough to get an in-depth analysis ( Vasileiou *et al.*, 2018).

**Figure 1A.**
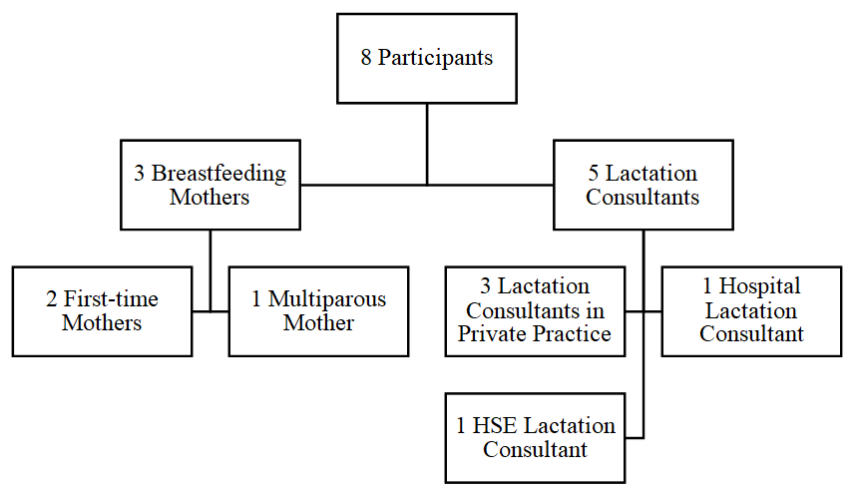
Flowchart of Participants Recruited.

A sample of three breastfeeding mothers was selected ( Table 1). Inclusion criteria involved being over eighteen years old and having given birth throughout the time that the COVID-19 restrictions were in place as these participants would be able to provide insights on their experience of breastfeeding support during the COVID-19 pandemic. Exclusion criteria therefore included being under 18 years old and not having experience of breastfeeding during the time that the COVID-19 restrictions were in place. Although previous studies looked specifically at primiparous women ( Alberdi *et al.*, 2018), for this study participants could partake regardless of whether they were first-time or multiparous mothers, as the context of COVID-19 is the focus here.

**Table 1.** Participant key.

Participant Key	
Lactation Consultant 1 (LC 1)	Health Service Executive (HSE) Lactation Consultant
Lactation Consultant 2 (LC 2)	Lactation Consultant in Private Practice
Lactation Consultant 3 (LC 3)	Lactation Consultant in Private Practice
Lactation Consultant 4 (LC 4)	Lactation Consultant in Private Practice
Lactation Consultant 5 (LC 5)	Hospital Lactation Consultant
Mother 1 (M 1)	Multiparous Mother
Mother 2 (M 2)	First-time Mother
Mother 3 (M 3)	First-time Mother

A sample of five lactation consultants was also selected ( Table 1). A lactation consultant can be defined as a healthcare professional that has specific training to deliver breastfeeding support and assist with the prevention, recognition and provision of solutions to breastfeeding difficulties ( Brooks *et al.*, 2016). The inclusion criteria involved being a an International Board Certified Lactation Consultant who provided breastfeeding support to mothers throughout the COVID-19 restrictions. Lactation consultants who were not actively providing support during the time that the COVID-19 restrictions were in place were excluded from this study.

An additional four breastfeeding mothers and five lactation consultants expressed interest in participating in this study, however they were lost to follow up and therefore did not participate in an interview ( Figure 1B). Given that this research was conducted as part of an undergraduate degree and given the time constraints of the project, there was no further attempt to recruit additional participants.

**Figure 1B.**
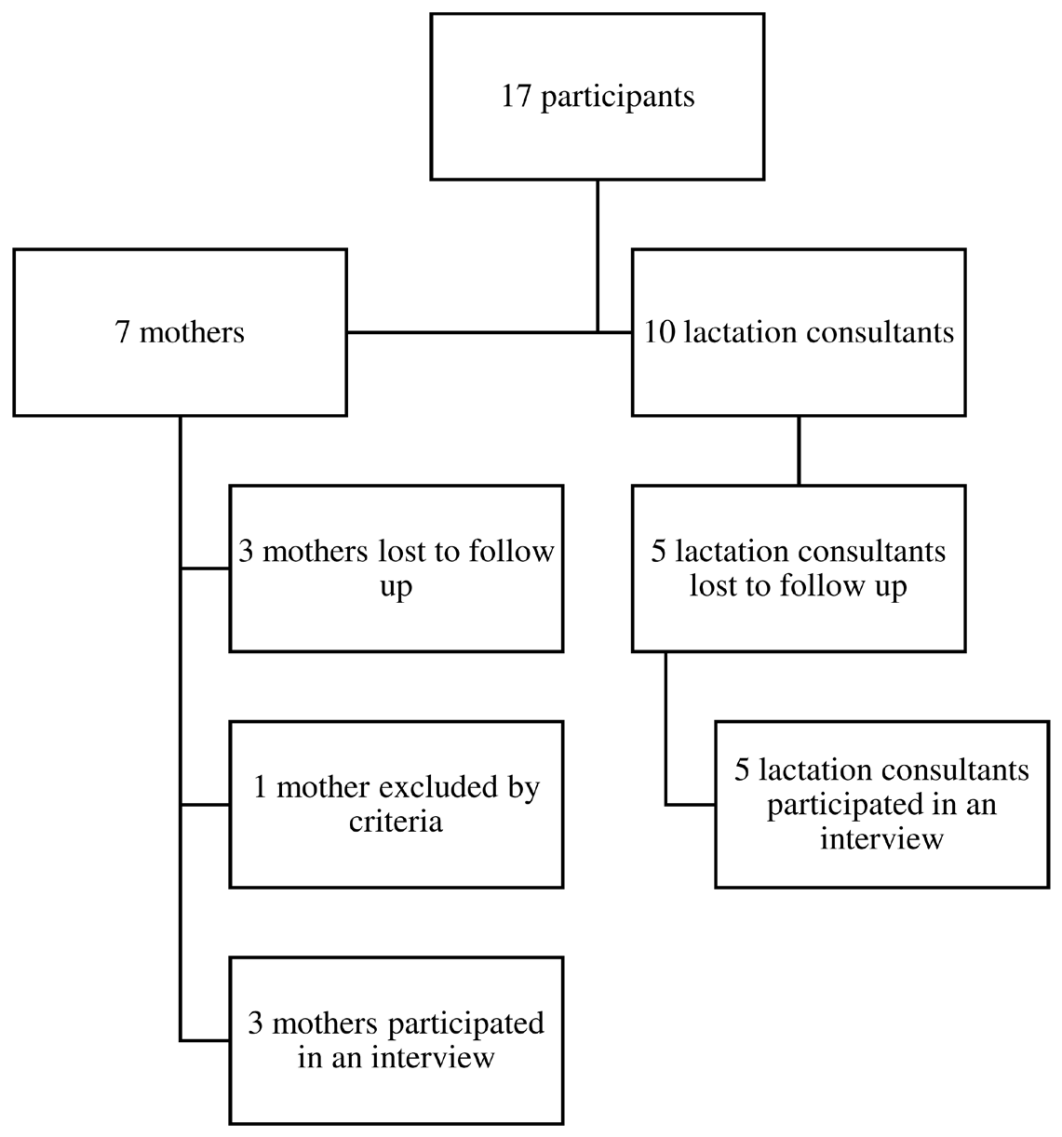
Flowchart of recruitment process.

### Instruments

Interview guides were developed by the researcher and used to conduct interviews (extended data). Based on review of the literature, the first author developed the interview guides with input from the second author. No demographic characteristics other beyond parity were collected during the interviews with the breastfeeding mothers.

### Ethical issues

This is a low-risk study, however consideration was given to ethical issues in qualitative research such as informed consent, privacy and confidentiality. This study involved the use of Zoom and the digital recording of interviews, therefore consideration was given to the digital data protection of participants ( Newman *et al.*, 2021). Ethical approval was sought and obtained from the Dublin City University Research Ethics Committee in November 2021.

### Procedure

Upon receiving ethical approval, permission was sought from the Association of Lactation Consultants in Ireland (ALCI) for an email to be distributed to members on behalf of the researcher. The researcher negotiated access and complied with the ALCI’s student research policy which required the submission of an application to be reviewed by five ALCI council members. On approval, an email was distributed. Interested participants could then email the researcher for further information. Recruitment of breastfeeding mothers involved emails and phone calls with a local PHN to identify best practices for identifying women to invite to participate in the study. As a result, breastfeeding support groups and local community groups were contacted and provided with information on the study via social media and interested participants were invited to follow up with the researcher to express their interest in participating in the study. Associate membership of the ALCI is accessible to individuals who are not IBCLCs but who have an interest in breastfeeding, therefore the email that was distributed to ALCI members outlined that both lactation consultants and breastfeeding mothers were invited to participate in interviews as part of this study. Again, interested breastfeeding mothers who received this email were invited to follow up with the researchers to express their interest and request further information in relation to participating. Plain language statements and informed consent forms were distributed via email and the interviews were scheduled. Informed consent forms were completed and returned prior to interviews taking place. The interviews were conducted by one researcher (AC) via Zoom as it is easy to access, user-friendly, has robust privacy settings, is time and cost efficient and is beneficial for participants in maintaining rapport with investigators ( Archibald *et al.*, 2019). The interviews, which took place in February 2022, lasted 30-40 minutes and were recorded using the Zoom audio-recording option. The audio-recorded interviews were then transcribed by one researcher (AC). The perspectives of breastfeeding mothers and lactation consultants were then analysed together using thematic analysis in order to capture both perceptions of the shared experience of breastfeeding support.

### Data analysis

Thematic analysis was chosen to analyse the collected data as it is used to identify, investigate and interpret patterns that are generated and constructed from the study of qualitative data throughout the research process ( Clarke & Braun, 2017). It is appropriate for this study given that it is used to understand thoughts, opinions, experiences and behaviours across data ( Kiger & Varpio, 2020). The patterns that are identified through thematic analysis are referred to as themes. They are developed from the generation of codes that encapsulate intriguing aspects of the data that may be relevant to the research question. Themes are the outcomes of thematic analysis that provide a framework for presenting and reporting on the researcher’s findings ( Clarke & Braun, 2017). Braun and Clarke’s (2006) six-step process for thematic analysis was used in this study to identify and interpret key aspects of the data that are relevant to the research question. The transcribed interviews were carefully read and codes were assigned to interesting features that were relevant to the research topic by one researcher (A.C). Patterns of similar codes emerged and from these patterns, common themes were generated. Each theme was then reviewed by both researchers and those that presented a strong pattern within the data were considered the most relevant. Any disagreements regarding the coding and theming were resolved by discussion between the two researchers. The themes were then assigned names based on the aspect of data that was covered and the insights it provided to the research topic. The themes were further developed to include sub-themes that provided a more detailed interpretation of the over-arching theme. Finally, a report was written up using themes to present the study’s findings. Extracts of data from the interviews that had been assigned codes and used to generate the themes were included in this write-up to put the themes into context and provide the reader with a raw illustration of the participants’ views, opinions and experiences.

Similar to the analysis approach used by Whelan and Kearney (2015), the categories that were developed from the coding of the transcripts were used to identify overarching themes from within both sets of interviews. The transcripts were considered separately and then combined as the overarching themes emerged from the thematic analysis. Given that staff and patient experiences are unequivocally intertwined in the delivery of good patient-centred care ( Ocloo *et al.*, 2020), it seemed appropriate to integrate data from the interviews with the mothers and the lactation consultants to gain an insight into both perspectives of the same phenomenon. This method of thematically analysing transcripts from interviews with both patients and healthcare professionals has previously been used by Maguire, Daffern and Martin (2014) to explore both patients’ and nurses’ perspectives of healthcare interventions.

## Findings

**Figure 2.**
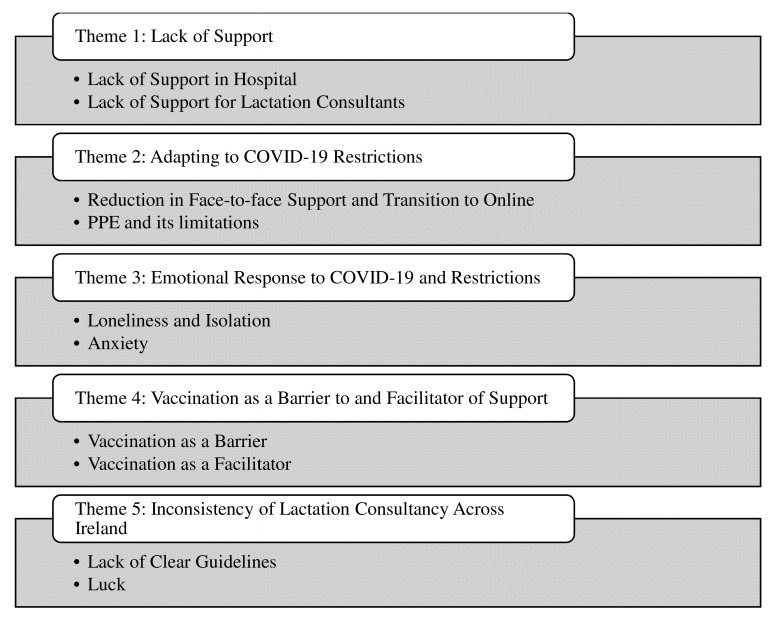
Themes Generated through Thematic Analysis.

The findings will be presented under themes that were identified through thematic analysis of the data collected. Both groups of participants who took part in this study identified lack of support as an integral part of their experience of lactation support throughout the COVID-19 restrictions. Both mothers and lactation consultants spoke of the adaptations that had to be made to the delivery of lactation consultancy throughout the pandemic. The mothers and lactation consultants also spoke about the emotions that they experienced in response to the COVID-19 restrictions during their own breastfeeding journeys or the journeys of mothers that they facilitated. Vaccination against SARS-CoV-2 as both a barrier and an enabler of breastfeeding support was also highlighted throughout the interviews in addition to the inconsistency of lactation consultancy throughout the restrictions. These themes are presented in Figure 2.

### Lack of support

The lack of support for both mothers and lactation consultants was highlighted by the participants throughout their interviews. A lack of support was particularly relevant amongst breastfeeding mothers in relation to the lack of access to lactation consultants in hospital settings and in the lack of face-to-face support groups. Although lack of support was a key feature of breastfeeding mothers’ experiences of lactation consultancy throughout the pandemic, lactation consultants within the HSE also felt they did not receive enough help in relation to Information Technology (IT) related support for moving their services to online platforms. Another interesting finding was the lack of supervision and support provided to lactation consultants, specifically in private practice where they do not work amongst a team of health professionals, to discuss the sometimes traumatic and distressing nature of their work.

***Lack of lactation consultancy and breastfeeding support in hospital settings.*** All the participants in this study identified the lack of breastfeeding support from lactation consultants within the hospital setting as a key feature of their experience of lactation consultancy throughout the pandemic:

“in those early stages they had no support, they were delivered and everybody kept away from each other” (LC 1)
  “I had no support from a lactation consultant or anything like that on the post-natal ward and again I found it quite unsupportive” (M 1)
  “I firmly believe, if my babies weren’t in NICU, they, I would not be breastfeeding … there was one hundred percent not adequate support on the post-natal ward” (M 1)

An notable finding was the suggestion from both mothers and lactation consultants that the issue of the unavailability of lactation consultants within hospital settings, although exacerbated by COVID-19, was an issue that existed pre-COVID-19:

“I mean you know this was even going back pre-COVID you know, the lack of support is shocking in hospitals like it really, really is” (LC 3)

An increase in mothers signaling their intention to breastfeed was highlighted however, according to the lactation consultants interviewed in this study, due to the lack of support available, many of these mothers will not have been able to fulfil that intention:

“the number of people signaling an intention to breastfeed will have gone up… but sadly too many of them will not have because of just the lack of available… it’s been fairly grim you know so people are not getting as much hands on support as they could do with” (LC 4)

The mothers who participated in this study spoke from their own experience of the lack of support they were offered in hospital and emphasised the importance of lactation consultancy by expressing their wishes to have had more support and time with a lactation consultant:

“I’d love if there was more support in the hospitals for new mums from professional lactation consultants… instead of a fleeting moment from a midwife who is under pressure you know and is trying her best (M 2)

The lactation consultants held the same view as the mothers about this topic and spoke of their experience of hearing stories from mothers that they have provided support to who were denied appointment from hospital lactation consultants as:

“she only comes to mothers with mastitis” (LC 3).

Frustration was also expressed by both lactation consultants and mothers at the limited hours that lactation consultants within hospitals are available for and the impact this has on the support given to and received by mothers.

“the lactation consultant at the time… was only there like two or three days a week” (M 1)
  “the lack of support is awful, I mean in any of the maternity hospitals the lactation consultant is nine to five Monday to Friday” (LC 3)

***Lack of support for lactation consultants.*** The lactation consultants in this study identified the lack of support available to them during their transition from face-to-face to online platforms as a key feature of their experience of delivering lactation consultancy throughout the COVID-19 restrictions. One HSE lactation consultant described the lack of IT support as a challenge they faced and another in public service spoke of having to work it out for themselves:

“IT support was very limited… I could have done with an awful lot more IT support” (LC 1)
  “[you] kind of work it out yourself” (LC 5)

Another finding in relation to support was the suggestion that lactation consultants, specifically those in private practice, do not have adequate support to help them cope with the sometimes-distressing nature of their work. The restrictions may have exacerbated the lack of support for lactation consultants by further limiting their contact with others. The need for a counsellor to facilitate debriefings and offer care to lactation consultants was identified:

“I just feel from the professional side of things we do need some kind of a minder or counsellor or debriefer” (LC 3)

### Adapting to COVID-19 restrictions

The COVID-19 restrictions forced lactation consultants to quickly adapt and alter the ways in which they delivered their support. Both mothers and lactation consultants had to turn to online platforms to continue support. These adaptations came easy to some, however others struggled and had difficulty getting used to them. PPE was worn to protect both mothers and babies and lactation consultants however, it didn’t come without its own issues.

***Reduction in face-to-face support and delivery of support via online platforms.*** All the participants in this study spoke of the reduction of face-to-face support due to the COVID-19 restrictions. Both mothers and lactation consultants had to adapt to the restrictions in order to continue delivering and receiving support. The lactation consultants adapted to the restrictions by offering their support through online platforms:

“I did an awful lot of, of Zoom or WhatsApp videos so we could see each… you know I had my knitted boob, I had my doll baby” (LC 3)

The mothers’ experiences of lactation consultancy via online platforms were varied. Several lactation consultants perceived that the mothers were satisfied with online support:

“Yeah, they love it and they stay in their bed” (LC 4)

The mothers themselves had varying experiences with online support. Some adapted well and were happy and grateful for it:

“I actually found doing it online really good like you know you kind of, you were in the comfort of your own home, you were in your own environment… we both found that really good” (M 2)

Others had difficulty adapting to the online world and didn’t place much value on online support:

“There was no local groups of that around, it all seemed to be online based…I didn’t really get much value from it” (M 3)
  “so many people… just wanted you face-to-face, they didn’t want zoom (LC 2)

Most lactation consultants adapted quickly to the online world and worked out ways to deliver their support through a screen:

“I would ask the mother to get a partner to video the baby coming to the breast or a close up of the baby latching or a video of the baby’s mouth when the baby is crying” (LC 3)

An interesting adaptation that mothers discussed was their reliance on social media for lactation consultancy and breastfeeding support throughout the Pandemic:

“I was always looking at lactation consultants on Instagram and like honest to God I learned more from them than anything else” (M 2)

***PPE and its limitations.*** The wearing of PPE was another adaptation that both lactation consultants and mothers had to make in order to continue face-to-face support, where that was done. Lactation consultants spoke about their experience of wearing gloves, aprons and masks and adapting to a new procedure regarding infection control practices that had to be carried out in between consultations:

“it was mask, gloves, apron and then as soon as you got home you got changed in an area of your home that wasn’t inside so either in the porch or you took off your clothes anyway and put them in the washing machine and changed and if you were going on to see a second client you had a full change of clothes and a shower” (LC 4)

Both mothers’ and lactation consultants’ experiences of lactation consultancy were impacted by communication issues due to wearing of PPE. Although all were grateful that the wearing of PPE facilitated the delivery of face-to-face support, they acknowledge that the wearing of masks had a huge impact on communication:

“wearing PPE, FFP2 masks… it’s difficult to communicate, difficult showing empathy” (LC 5)

Physical contact is an integral part of lactation consultancy however, adhering to social distancing rules had a huge impact on the personal relationship and interactions between mothers and lactation consultants which undoubtedly had a negative impact on both parties’ experience of lactation consultancy:

“not being able to hug somebody, place a hand on their shoulder, shake hands with them, all the things as human beings that we do to interact and build up a relationship in a very short period of time” (LC 5)

### Emotional response to covid-19 and restrictions

The COVID-19 restrictions appear to have had a huge impact on the emotions that women felt throughout their experience of lactation consultancy. Social distancing and travel restrictions saw them unable to see family, friends and other mothers and limited their access to lactation consultancy. These restrictions resulted in feelings of loneliness, isolation and anxiety. 

***Loneliness and isolation.*** Both mothers themselves and lactation consultants spoke about the experience of loneliness and isolation throughout the restrictions. The mothers spoke about their experience of feeling isolated by not having support groups where they could meet other mothers and be shown breastfeeding techniques in-person. The COVID-19 restrictions isolated them by taking away their opportunity to ask questions, share stories and have their feelings and experiences normalized by conversations with other mothers:

“I think the isolation of not having support group to just pop in on a weekly basis and say hi, I’m a new mum, I’m struggling” (M 3)

Lactation consultants spoke from their experience of meeting women who felt abandoned by the lack of support available, resulting in them feeling very alone, abandoned and vulnerable:

“they were very vulnerable, they were very, very alone” (LC 3)

***Anxiety.*** Most lactation consultants spoke about the increased levels of anxiety in women who have had babies throughout the pandemic. Mothers also reported feeling anxious and scared due to COVID-19 and the restrictions:

“I’ve seen a general level of anxiety… around birth and some fear for COVID as well” (LC 4)
  “it was all on you and you were a first-time mam on your own and you know that was scary” (Mum 2)

### Vaccination against SARS-CoV-2 as a barrier to and facilitator of support

An interesting finding identified through this research was the impact that vaccination status had on access to support in certain areas. Several lactation consultants discussed vaccination against SARS-CoV-2 as both a barrier to and facilitator of support. Although immunity was discussed by both lactation consultants and mothers, vaccination in relation to access to breastfeeding support was only discussed among the lactation consultants who participated in the research study.

***Vaccination against SARS-CoV-2 as a barrier.*** One lactation consultant spoke about her experience of delivering lactation consultant led support groups. Once the support group had resumed in-person, there were, and still are, certain guidelines that must be adhered to. She spoke about vaccination status as an indicator of ability to access the breastfeeding support group. Only mothers who were fully vaccinated could attend the support groups:

“people who weren’t vaccinated weren’t allowed to come to breastfeeding support group and still aren’t” (LC 1)

Although those who were not vaccinated against the SARS-CoV-2 virus were not allowed to attend the support groups, they were not denied access to a lactation consultant. The lactation consultant would contact the unvaccinated person and offer them a one-on-one consultation to discuss any issues and provide them with support:

“I couldn’t have her at the breastfeeding support group, what I did do was tell her I’d see her on a one-to-one” (LC 1)

***Vaccination against SARS-CoV-2 as a facilitator.*** Several lactation consultants described their experience of returning to face-to-face lactation consultancy once they had been vaccinated against the SARS-Cov-2 virus. Vaccination, in this case, acted as an enabler to resuming face-to-face support. Those who were at high-risk due to underlying health conditions who did not feel comfortable offering support face-to-face were able to remain online until they received their SARS-CoV-2 vaccination, at which stage they could then decide if they felt comfortable to return to face-to-face support:

“…because I’m diabetic I chose not to do any face-to-face until we were vaccinated so I did all my client calls by Zoom… once I’d had both my vaccines I started going back out” (LC 4)

### Inconsistency of lactation support across Ireland

***Lack of clear guidelines.*** There did not appear to be a standardised protocol in relation to the delivery of lactation consultancy throughout the COVID-19 restrictions. There appear to have been geographical discrepancies in relation to what lactation consultants were authorised to do. Lactation consultancy was deemed an essential service in some areas and therefore in-person, face-to-face support continued, however in other areas face-to-face support was paused and only online support could be delivered. This variation suggests that there was no single cohesive or standardised approach to breastfeeding support throughout the pandemic:

“in some parts of the country the support was non-existent… they weren’t allowed see people” (LC 1)

***Luck.*** Both mothers and lactation consultants discussed the lack of consistency within support for breastfeeding mothers, particularly in hospital. Mothers described their experiences of getting support by chance or because they were lucky that a midwife with a specific interest in breastfeeding was on that shift:

“it would have depended on the day they had their baby, the day they were discharged and whether or not the staff were overrun… if they happened to find the right person on the right day, absolutely they’ll have had lots of help and support but if they didn’t then they mightn’t have had what they needed” (LC 4)

It is evident that both mothers’ and lactation consultants’ experiences of lactation consultancy have been hugely impacted by the restrictions put in place due to COVID-19, however it is clear that these services were already limited before the pandemic. These findings have highlighted the considerable impact COVID-19 had on mothers’ access to support and how the restrictions impacted the way in which breastfeeding support was delivered. The findings have also demonstrated the various adaptations which had to be made to the delivery of lactation consultancy and how these changes impacted mothers’ experiences of lactation consultancy.

## Discussion and conclusions

The provision of high quality, timely and effective breastfeeding support remains a challenge, which has been exacerbated by COVID-19, in Ireland and globally. The findings of this research suggest that some mothers have inadequate breastfeeding support in hospitals with little or no access to lactation consultants. The DoH (2016) previously identified this issue, therefore suggesting that although exacerbated by COVID-19, it pre-existed. Both lactation consultants and mothers in this study expressed their views that these issues existed pre-COVID-19. Similar views on this topic have been expressed previously ( Whelan & Kearney, 2015), which strengthens the evidence that this is not a novel issue. This research presented an interesting finding on the varied experiences that women had of online lactation consultancy. Whilst some mothers struggled with insufficient online consultations, similar to those in a study conducted by Panda *et al.* (2021), others enjoyed and were satisfied with the online support.

Another important finding was the lack of support available to lactation consultants. Healthcare professionals who worked throughout COVID-19 are at a higher risk of mental health problems, therefore it seems necessary to provide better psychosocial support for them ( Stuijfzand *et al.*, 2020). Previous research suggests that clinical debriefing for healthcare workers who experience trauma at work can be useful in reducing post-traumatic stress symptoms ( Scott *et al.*, 2022). These findings provide rationale for the implementation of clinical debriefings into lactation consultants’ routines.

Adaptations have evidently had to be made to the delivery of lactation consultancy throughout the COVID-19 restrictions. The transition from in-person to virtual support is a significant adaptation that has been previously identified ( Brown & Shenker, 2021; Panda *et al.*, 2021). Both mothers and lactation consultants have had to adapt to reduced face-to-face support ( Kinoshita & Doolan, 2021; Panda *et al.*, 2021), an aspect of breastfeeding support shown to be valuable to mothers ( Ingram *et al.*, 2020) and successful in encouraging women to continue and exclusively breastfeed ( Gavine *et al.*, 2022; World Health Organization, 2018). Another significant adaptation to the delivery of lactation consultancy was the wearing of PPE. Both mothers and lactation consultants had difficulty adapting to this change due to issues communicating with masks. This difficulty has been previously mentioned by Green *et al.* (2021) and raises an interesting concern going forward as to whether in-person support with the use of masks is more beneficial than virtual consultations.

Lactation consultants discussed the loneliness, isolation and anxiety experienced by breastfeeding mothers in response to COVID-19. The mothers themselves agreed that due to the lack of support from healthcare professionals, family and friends and the unavailability of support groups, they felt alone and isolated. Some mothers expressed their anxiety about contracting COVID-19 and their babies becoming ill. Feelings of loneliness, isolation and anxiety, as expressed by both lactation consultants’ perceptions of mothers’ emotions and mothers’ descriptions of their own emotions, has been previously identified ( Jackson *et al.*, 2021; Kotlar *et al.*, 2021; Pacheco *et al.*, 2021; Sakalidis *et al.*, 2021). This highlights the necessity of providing support to mothers to protect their well-being.

Vaccination against SARS-CoV-2 as a barrier and facilitator to accessing and providing support was an interesting finding. Healthcare workers, who work in close proximity to people, are at an increased risk of exposure to COVID-19 ( Dooling *et al.*, 2020) and group gatherings are a risk factor for contracting COVID-19 ( Rashedi *et al.*, 2020). This provides rationale for compulsory vaccination in order to attend support groups for the protection of others and those unvaccinated. Although compulsory vaccination can worsen inequities in access to resources ( Omer *et al.*, 2019) such as support, that is not the case here as support was offered on a one-to-one basis regardless of vaccination status. However, the emotional support networks that breastfeeding support groups provide is widely acknowledged therefore, mothers that could not attend support groups may have missed out on important socialisation and informal peer support. Similar experiences have been identified by Snyder and Worlton (2021) who reported on the desire of mothers to have peer support after missing their weekly breastfeeding support groups due to COVID-19.

Some lactation consultants felt safe and protected once they had received their SARS-CoV-2 vaccinations and this enabled them to resume face-to-face support. Previous studies show that other healthcare professionals also felt that the COVID-19 vaccines would protect them and their families from contracting the infection ( Al-Metwali *et al.*, 2021) therefore, suggesting that vaccination facilitated them to feel safe and protected whilst at work.

Inconsistency of lactation support across Ireland due to the lack of standardised protocol for all lactation consultants has resulted in women having varying experiences of accessing support. Although global best practice guidelines for the provision of support to women for improving breastfeeding practices have been published by the World Health Organization (2018), it is clear that the best practices outlined in these guidelines may not be implemented. Inconsistency in the application of restrictions has also previously been identified on a European level, with guidelines from authoritative bodies such as the WHO being adhered to at varying degrees by individual countries and specific areas within countries ( Lalor *et al.*, 2021). The lack of a cohesive approach to lactation consultancy in Ireland throughout the pandemic has resulted in unequal distribution of support which may have subsequently led some women to cease breastfeeding early or be unable to fulfil their wish to breastfeed.

### Limitations

The small sample size of this study, appropriate for a qualitative study, means that the findings are not generalisable. Linked interviews conducted with patients and healthcare professionals can potentially provide a deeper understanding of needs and experiences than interviews that explore single perspectives. These types of multi-perspective interviews provide opportunities for exploring similarities and differences in the perceptions of and integrating service improvement suggestions from both patients and healthcare professionals ( Kendall *et al.*, 2009). In this study, it was not possible to recruit participant matches in terms of lactation consultant and the mothers they supported. Due to this limitation, similar to findings from Whelan and Kearney (2015), the findings from both groups cannot be directly correlated to each other. Despite this limitation, this study may contribute important information in relation to breastfeeding support by placing mothers’ views about support in the context of their experiences and integrating them with the views of those delivering support to provide practical recommendations for the improvement of such services. Although both private and public lactation consultants were interviewed, it was not possible to get an equal representation from each sector. Fewer mothers than lactation consultants participated in this study, nevertheless including both perspectives in this small study was seen to be of value.

### Implications of the research

The findings of this study suggest that although the COVID-19 restrictions played a role in the limitation of services available to mothers, it is evident that the issue regarding the lack of lactation consultants in hospital settings existed before COVID-19. More research should be undertaken to investigate why there are so few lactation consultants available in hospitals. Research investigating attitudes to breastfeeding in medical settings could be beneficial in identifying why breastfeeding does not appear to be sufficiently supported in hospital settings. Further research may also be warranted to identify how lactation consultancy should be managed in future emergency situations. This research suggests a need for support for lactation consultants, specifically in private practice given that they often work alone, to allow them to discuss and share the burden of the sometimes-distressing nature of their work, through clinical supervision or peer-support or other mechanisms. In the context of future emergency situations, this research supports an approach that would deem lactation consultancy and breastfeeding support an essential service. Any new policy should be accompanied by a monitoring framework setting out clear actions and indicators to effectively monitor implementation and progress in this area.

## Data Availability

The data used to inform this research was collected via interviews with participants who provided their informed consent. The interviews were recorded and transcribed however, these datasets are not publicly available due to participants being informed at the time of giving consent that the data collected would not be accessed by others and would be destroyed after one year. The interview guides used to conduct the interviews in this study have been included as extended data. A file titled ‘Extended data - Interview Guides’ has been uploaded to Open Science Framework data repository. DOI 10.17605/OSF.IO/Q9M3N. License: *CC0 1.0 Universal*
