# Supplementary material for: Exploring Breastfeeding Mothers’ and Lactation Consultants’ Experiences of Lactation Consultancy Throughout the Restrictions Put in Place Due to the COVID-19 Pandemic
Source: HRB Open Res. 2024 Dec 16;7:9. Originally published 2024 Feb 19. [Version 2] doi: 10.12688/hrbopenres.13856.2 (PMC11757925; doi:10.12688/hrbopenres.13856.2)
Supplement: Peer review history

# Reviewer report: Reviewer response for version 2

**DOI:** 10.21956/hrbopenres.15421.r45101

**Published:** 2025-02-13

**Authors:** Charlotte M Wright

**Affiliations:** 1 University of Glasgow, Glasgow, UK

This modest  qualitative study has already been extensively reviewed and amendments made. The question addressed was fairly limited and the numbers studied small, but it seems to be appropriately written up and i have no substantive comments to make apart from  a minor issue in the introduction, where the authors refer to only 15% Irish babies being exclusively breast feed at age 6 months. This is not a helpful age to work with, as  mothers are advised to start solids at around 6 months. Are they sure that this figure doesn't in fact reflect the proportion of all children aged <6m who are exclusively breastfed?

Is the work clearly and accurately presented and does it cite the current literature?

Yes

If applicable, is the statistical analysis and its interpretation appropriate?

Not applicable

Are all the source data underlying the results available to ensure full reproducibility?

No

Is the study design appropriate and is the work technically sound?

Yes

Are the conclusions drawn adequately supported by the results?

Yes

Are sufficient details of methods and analysis provided to allow replication by others?

Yes

Reviewer Expertise:

Infant and child nutrition

I confirm that I have read this submission and believe that I have an appropriate level of expertise to confirm that it is of an acceptable scientific standard.

# Reviewer report: Reviewer response for version 2

**DOI:** 10.21956/hrbopenres.15421.r44238

**Published:** 2025-02-03

**Authors:** Kathleen Kendall-Tackett

**Affiliations:** 1 Texas Tech University Health Sciences Center, Amarillo, USA

This article considers the subject of COVID restrictions on breastfeeding in Ireland, noting that Ireland has one of the lowest exclusive breastfeeding rates in Europe prior to COVID. 

 I note that this is a revised article, but this is my first time seeing it. 

 Introduction

 Overall, the writing is clear. However, I found the structure of the introduction confusing. The focus is on the impact of COVID on breastfeeding rates. Yet that topic is only in the third paragraph. 

 I'm not sure that the authors need to describe the benefits of breastfeeding (para 1) and the importance of lactation consultants (para 2) before they describe what they are actually studying (para 3). I recommend that they narrow the focus to COVID and bring in the already low rates in Ireland and how COVID made it worse for both LCs and mothers. That's the focus. The other information takes away from that but can be briefly summarized once the authors make clear that their focus is breastfeeding during COVID.

 Methods

 I recommend moving the objectives to the Introduction. The purpose of the intro is to justify the study: tell us why it is necessary. That section should end with laying out the objectives of the study.

 Why is the impact of COVID on LCs important? Yes, they were definitely affected, but how does that impact the breastfeeding rates? Try to make that connection clearer while focusing on *rates during COVID.*

 When describing the qualitative portion, the authors really do not need to describe why qualitative studies are necessary. People pretty much get that. I would like to see how they plan to collect and analyze their data instead. What method will/did they apply?

 What is the "target population"? That is very vague. Are they local? Are they online? Are they all Irish? We need to know that so we can understand where you selected your participants from.

 The authors do not need to editorialize about the "rich data" the participants will provide. We assume that. Just tell us more about who they are and how they were selected.

 We need much more information about the interview guides. How were the questions selected? What was asked? The idea is to provide enough info that someone could replicate the study.

 The authors can cut the explanation about Zoom. Most of us are familiar with it. We don't need to justification for it.

 Results

 It's confusing to have the LC comments mixed with the mothers' comments. I recommend separating them but noting their similarity with a transition sentence.

 I'm not clear about why LCs felt unsupported beyond not knowing how to use Zoom. And it sounds like many LCs were pleased during this time. It isn't very clear through this section.

 As for the other effects, they are what others in the very large lit on COVID are saying. Loneliness and isolation are definitely not unique. Please make it clear why this is important and unique for breastfeeding mothers and their helpers.

 There have been numerous studies on the effects of COVID on healthcare providers. It would strengthen this article if the authors included some of those and then noted why their sample was unique. It's been so bad here that some LCs have started special support groups for mothers who gave birth during COVID. It took a wrecking ball through maternal mental health that has been well-documented. Therefore, the authors' task is the show why their study is necessary and unique. In its current form, I am not getting much of that from this article, but it could be improved.

 Discussion

 I also would not call a small sample for a qualitative study a limitation. It's expected that the sample is small. 

 When discussing the implications, the authors have not made the case that COVID made things more difficult. It sounds as if they are already bad. How did COVID make it worse? I know it's challenging in Ireland; I have several Irish LC colleagues. 

 I am happy that you have done this study, but we need a lot more information from the authors.

Is the work clearly and accurately presented and does it cite the current literature?

Partly

If applicable, is the statistical analysis and its interpretation appropriate?

No

Are all the source data underlying the results available to ensure full reproducibility?

Yes

Is the study design appropriate and is the work technically sound?

Partly

Are the conclusions drawn adequately supported by the results?

Partly

Are sufficient details of methods and analysis provided to allow replication by others?

No

Reviewer Expertise:

Maternjal mental health, psychology, lactation, IBCLC, psychological trauma

I confirm that I have read this submission and believe that I have an appropriate level of expertise to confirm that it is of an acceptable scientific standard, however I have significant reservations, as outlined above.

# Reviewer report: Reviewer response for version 2

**DOI:** 10.21956/hrbopenres.15421.r44208

**Published:** 2025-01-23

**Authors:** Jane Francis

**Affiliations:** 1 Acadia University, Wolfville, NS, Canada

I have reviewed the revised manuscript and amend my previous status of Approved with Reservations to Approved. However, there are two corrections needed in the abstract and introduction:

- It appears the conclusion still states “Increased availability of lactation consultants and the implementation of breastfeeding rooms within hospitals is required”, but the main body of the text does not mention or discuss “breastfeeding rooms” in hospital. I would recommend removing this statement so that conclusions align with the presented findings.
- The sentence defining the role of the IBCLC remains in the introduction section of the manuscript and should be removed.

Is the work clearly and accurately presented and does it cite the current literature?

Partly

If applicable, is the statistical analysis and its interpretation appropriate?

Not applicable

Are all the source data underlying the results available to ensure full reproducibility?

No

Is the study design appropriate and is the work technically sound?

Yes

Are the conclusions drawn adequately supported by the results?

Partly

Are sufficient details of methods and analysis provided to allow replication by others?

Partly

Reviewer Expertise:

NA

I confirm that I have read this submission and believe that I have an appropriate level of expertise to confirm that it is of an acceptable scientific standard.

# Reviewer report: Reviewer response for version 1

**DOI:** 10.21956/hrbopenres.15184.r39463

**Published:** 2024-06-11

**Authors:** Jane Francis

**Affiliations:** 1 Acadia University, Wolfville, NS, Canada

Thank you for the opportunity to review the manuscript “Exploring breastfeeding mothers’ and lactation consultants’ experiences of lactation consultancy throughout the restrictions put in place due to the COVID-19 pandemic”. Using semi-structured interviews, the aim of the study was to explore breastfeeding mothers’ and lactation consultants’ experiences of breastfeeding support throughout COVID-19 pandemic restrictions in Ireland. It is important to speak to those receiving and providing breastfeeding support services in order to better understand how support can be implemented within specific contexts. This is a well-written manuscript which could be strengthened by addressing the comments below.

**Abstract**

 The conclusion states “Increased availability of lactation consultants and the implementation of breastfeeding rooms within hospitals is required”, but the main body of the text does not mention or discuss “breastfeeding rooms” in hospital. I would recommend removing this statement so that conclusions align with the presented findings.

**Introduction**

 The McFadden et al 2017 Cochrane review has been updated, therefore, the following reference should be included throughout the paper: (Gavine A, et al., 2022 [Ref 1]). Another reference which would be important to include in the paper would be the WHO guidelines entitled “Counselling of Women to Improve Breastfeeding Practices" ( https://www.who.int/publications/i/item/9789241550468) to highlight that global best practice guidelines do exist but may not be implemented (as noted by the participants in this study). This reference may fit well into the discussion.

 The sentence “The rate of babies that had received any breastfeeding on discharge from hospital dropped 4.9% between 2019 and 2020 in Ireland. Only 58.5% of babies had received any type of breastfeeding on discharge from hospital in 2020 in comparison to 63.4% in 2019” should be revised to indicate “…from hospital dropped 4.9 percentage points” instead of 4.9 percent.

 In the introduction, the sentence “This qualitative research study aims to explore both breastfeeding mothers’ and lactation consultants’ experiences of receiving and giving breastfeeding support throughout the restrictions put in place in Ireland due to the COVID-19 pandemic” can be removed since it’s in the middle of the introduction, or it can be moved to the last paragraph of the introduction (although the high-level aim is already noted at the end of the introduction and specific aims and objectives are in the methods section).

 In the introduction, the sentence “The term lactation consultant will be frequently referred to. A lactation consultant can be defined as a healthcare professional that has specific training to deliver breastfeeding support and assist with the prevention, recognition and provision of solutions to breastfeeding difficulties (Brooks et al., 2016)” seems a bit out of place. It may fit better in the methods when describing IBCLCs, which was the specific profession of focus for this study.

**Methods**

 Instruments/interview guide: was the interview guide developed by both authors or just one of the authors? It would be helpful to indicate who was involved in instrument development and consider including a sample of interview questions in a table or the complete set of questions as an appendix.

 Sample: were there any exclusion criteria?

 Ethical issues: it would be helpful to spell out the DCU acronym.

 Under Procedure, the sentence states “Recruitment of breastfeeding mothers involved emails and phone calls with ae local PHN, breastfeeding support groups and community groups”. More information is needed on the recruitment process. For example, how were these relationships established/how were the PHN, breastfeeding support groups and community groups chosen? Did the PHN and/or group staff and/or researcher approach specific women who might be interested or were all women/clients in a specific time period provided with the study information? Were women provided with information on the study and then required to follow up with the researcher if they were interested in participating?

 Under data analysis, the sentence states “This method of thematically analysing transcripts from interviews with both patients and healthcare professionals has previously been used by to explore both patients’ and nurses’ perspectives of healthcare interventions.” It would be helpful in the procedure section for sample size justification that the intent was to analyze perspectives of mothers and IBCLCs together.

 Under data analysis, the sentence states “Thematic analysis was chosen to analyse the collected data as it is used to identify, investigate and interpret patterns that emerge from datasets.” Braun and Clarke argue that themes do not emerge from data (Braun V, et al., 2022 [Ref 2]). Given Braun and Clarke’s thematic analysis process was used, different wording could be used here.

**Findings**

 Some clarity on the sample could be included. For example, were the 8 participants only those who responded to researchers or were there others who were approached/expressed interest who did not end up participating (e.g., an extension of figure 1 could be done to indicate if anyone was approached and indicated interest, but was lost to follow up). Was there any attempt to recruit more mothers or lactation consultants for the study?

 Confirm demographic characteristics were not collected from participants beyond parity among the mothers. This could be stated in the methods.

 Was the theme ‘vaccination as a barrier to and facilitator of support’ only discussed amongst LCs and not mothers? If so, it would be a good idea to make a note of this explicitly in the findings since the interviews from both mothers and IBCLCs were analyzed together.

**Discussion**

 In the discussion, it states “Although compulsory vaccination can worsen inequities in access to resources (Omer et al., 2019) such as support, that is not the case here as support was offered on a one-to-one basis regardless of vaccination status.” However, unvaccinated mothers could not attend group sessions, which may be important for informal peer support and socialization.

**Implication of findings**

 Under implication of findings, the sentence “Research investigating attitudes to breastfeeding in medical settings could be beneficial in identifying why breastfeeding does not appear to be sufficiently encouraged in hospital settings” does not appear to be supported from the findings of this study. The word “encouraged” could be changed to “support” to better align with the study findings.

**Editorial errors:**

- Quotation marks missing for some themes in abstract
- “ae” under procedure on page 5 should be “a”
- (A.C) on page 5 should be “A.C.”
- “saw then” should be “saw them” on page 8

Is the work clearly and accurately presented and does it cite the current literature?

Partly

If applicable, is the statistical analysis and its interpretation appropriate?

Not applicable

Are all the source data underlying the results available to ensure full reproducibility?

No

Is the study design appropriate and is the work technically sound?

Yes

Are the conclusions drawn adequately supported by the results?

Partly

Are sufficient details of methods and analysis provided to allow replication by others?

Partly

Reviewer Expertise:

infant and maternal nutrition, breastfeeding support, food insecurity, community nutrition

I confirm that I have read this submission and believe that I have an appropriate level of expertise to confirm that it is of an acceptable scientific standard, however I have significant reservations, as outlined above.

## References

[1] : Support for healthy breastfeeding mothers with healthy term babies. *Cochrane Database Syst Rev* .2022;10(10) : 10.1002/14651858.CD001141.pub6 CD001141 10.1002/14651858.CD001141.pub6 36282618 PMC9595242

[2] : Toward good practice in thematic analysis: Avoiding common problems and be(com)ing aknowing researcher. *International Journal of Transgender Health* .2023;24(1) : 10.1080/26895269.2022.2129597 1-6 10.1080/26895269.2022.2129597 PMC987916736713144

# Response

**Published:** 2024-12-05

**Authors:** Anna Connolly

**Affiliations:** Dublin City University, Ireland

We would like to thank the reviewer for their support of our work and constructive feedback on our manuscript.

 The abstract has been amended to ensure that the conclusions align with the presented findings and the statement in relation to breastfeeding rooms has been removed. The full interview guides have now been included in the appendix of the paper.

 In light of the update to the McFadden et al 2017 Cochrane review, the reference Gavine A, et al., 2022 has been included throughout the manuscript. Reference to the WHO guidelines entitled “Counselling of Women to Improve Breastfeeding Practices" has also been made in the discussion section of the manuscript to acknowledge that guidelines in relation to best practices exist but may not be implemented. Furthermore, the sentence in relation to the rates of breastfeeding has been amended to state that “The rate of babies that had received any breastfeeding on discharge from hospital dropped 4.9 percentage points between 2019 and 2020 in Ireland”. On review of the manuscript, the sentence stating the aim of the research in the middle of the introduction section was deemed superfluous and has been removed. The sentence defining the role of the IBCLC has been removed from the introduction section of the manuscript and inserted into the methods section.

 The interview guides were developed from the literature by the first author and with input from the second author.

 The manuscript has been amended to include any exclusion criteria in relation to the sample of mothers and lactation consultants in this study. In relation to the ethical issues, Dublin City University has been included in the manuscript to spell of the DCU acronym. Under the procedure section, the sampling strategy and recruitment process has been amended to provide further clarity on how the participants were sourced for this study. All potential participants were provided with information on the study and were then required to follow up with the researcher via email to express their interest in participating. Under the data analysis section, it has been clarified for sample size justification that the intent was to analyse the perspectives of mothers and IBCLCs together. Furthermore, this section has also been amended to correctly reflect Braun and Clarke’s thematic analysis process and clarify that the themes were generated or constructed through the analysis of the qualitative data collected through the interviews.

 Further clarity has been provided in relation to the sample. Figure 1 has been updated to demonstrate the additional participants that expressed interested but were lost to follow up. Given the time constraints due to this research being conducted as part of an undergraduate degree, there were no further attempts to recruit additional mothers or lactation consultants for participation in this research. The methods section has been amended to provide confirmation that demographic characteristics beyond parity were not collected among the mother participants. Furthermore, the findings section has been amended to clarify that the theme ‘vaccination as a barrier to and facilitator of support’ was only discussed amongst LCs.

 In the discussion section, the impact of not being able to attend breastfeeding support groups on informal peer support and socialisation has been acknowledged. Under the implication section, the word “encouraged” has been changed to “supported” in order to ensure that the implications align with the study findings.

 Finally, the editorial errors outlined in the review have been corrected in the manuscript.

 Thank you again for your comments, feedback and support of this research.

# Reviewer report: Reviewer response for version 1

**DOI:** 10.21956/hrbopenres.15184.r39009

**Published:** 2024-05-07

**Authors:** Louise Gallagher

**Affiliations:** 1 Trinity College Dublin, Dublin, Ireland

Review:

 The aim of this study was to explore breastfeeding mothers’ and lactation consultants’ experiences of breastfeeding support throughout the COVID-19 restrictions in Ireland. support.

 This was overall a well written piece of work and I commend the student for this important and rigorously conducted study.

 On page 4 it states that LC’s should be fully qualified and registered with ALCI. This needs to be reworded as ALCI is not a registration body for IBCLC’s and has both affiliate (certified) and non-affiliate (uncertified or lay) members.

 Although the sample is identified as ‘purposive’ there is a lack of clarity on how participants were sourced. It’s unclear if this has any impact or introduces any inherent biases but this requires further detail.

 The use of the term ‘unanticipated’ throughout the findings suggests some degree of bias that many of the findings were expected. While this may indeed be the case it comes across as somewhat biased in parts, and I would suggest rephrasing in relation to some of the findings. 

 The ‘Vaccination as a barrier’ should include SARS- CoV-2 as part of the title as I read this earlier in the paper to be related infant vaccination.

 The findings (line 2) should clarify that ‘some mothers have inadequate breastfeeding support in hospitals with little or no access to lactation consultants’. The current statement presents some overreach given the small sample size of mothers and LC’s.

 Typos:

 Page 5 line 10 has a redundant e before ‘a local PHN’.

 Review initials for consistency (sometimes AC and also A.C)

Is the work clearly and accurately presented and does it cite the current literature?

Yes

If applicable, is the statistical analysis and its interpretation appropriate?

Not applicable

Are all the source data underlying the results available to ensure full reproducibility?

Yes

Is the study design appropriate and is the work technically sound?

Yes

Are the conclusions drawn adequately supported by the results?

Yes

Are sufficient details of methods and analysis provided to allow replication by others?

Partly

Reviewer Expertise:

Breastfeeding, mixed methods, maternity care

I confirm that I have read this submission and believe that I have an appropriate level of expertise to confirm that it is of an acceptable scientific standard.

# Response

**Published:** 2024-12-05

**Authors:** Anna Connolly

**Affiliations:** Dublin City University, Ireland

We would like to thank the reviewer for their support of our work and constructive feedback on our manuscript.

 The manuscript has been amended on page four to clarify that the inclusion criteria for participating in the interviews involved being an International Board Certified Lactation Consultant rather than registered with the ALCI.  

 Furthermore, the sampling strategy and recruitment process has been amended to provide further clarity on how the participants were sourced for this study.

 The sections of the manuscript that referred to unanticipated findings have been rephrased to reduce any suggestions of bias.

 The manuscript has also been amended to include SARS-CoV-2 as part of the title for the findings related to vaccination.

 In light of the comments in relation to the overreach of the statement in line 2 of the discussion section given the small sample size, the manuscript has been amended to clarify that ‘some mothers have inadequate breastfeeding support in hospitals with little or no access to lactation consultants.

 Finally, the typos on page 5 and the inconsistencies in relation to the authors initials throughout the manuscript have been corrected.

 Thank you again for your comments, feedback and support of this research.
